# The Immune System Drives Synapse Loss During Lipopolysaccharide-Induced Learning and Memory Impairment in Mice

**DOI:** 10.3389/fnagi.2019.00279

**Published:** 2019-11-15

**Authors:** Yi-Rong Xin, Jun-Xing Jiang, Yang Hu, Jun-Ping Pan, Xiang-Nan Mi, Qin Gao, Fei Xiao, Wei Zhang, Huan-Min Luo

**Affiliations:** ^1^Department of Pharmacology, College of Basic Medicine, Jinan University, Guangzhou, China; ^2^Institute of Brain Sciences, Jinan University, Guangzhou, China; ^3^Department of Parasitology, Ningxia Medical University, Yinchuan, China

**Keywords:** lipopolysaccharide, learning and memory impairment, neuroinflammation, complement, microglia

## Abstract

Although lipopolysaccharides (LPS) have been used to establish animal models of memory loss akin to what is observed in Alzheimer’s disease (AD), the exact mechanisms involved have not been substantiated. In this study, we established an animal model of learning and memory impairment induced by LPS and explored the biological processes and pathways involved. Mice were continuously intraperitoneally injected with LPS for 7 days. Learning- and memory-related behavioral performance and the pathological processes involved were assessed using the Morris water maze test and immunostaining, respectively. We detected comprehensive expression of C1q, C3, microglia, and their regulatory cytokines in the hippocampus. After 7 days of LPS administration, we were able to observe LPS-induced learning and memory impairment in the mice, which was attributed to neural impairment and synapse loss in the hippocampus. We elucidated that the immune system was activated, with the classical complement pathway and microglial phagocytosis being involved in the synapse loss. This study demonstrates that an LPS-injected mouse can serve as an early memory impairment model for studies on anti-AD drugs.

## Introduction

Alzheimer’s disease (AD) constitutes most of the dementia cases, affecting 10% of the population aged over 65 years and 50% of those aged over 85 years (Hebert et al., 2003). Currently, there are more than 20 million AD patients worldwide. Furthermore, with the increasing aging population, the morbidity, and mortality rates have increased year by year (Alzheimer’s Association, 2012). AD seriously jeopardizes the quality of life of elderly individuals and increases the burden on their guardians and society. AD is pathologically characterized by neuron loss, neuroinflammation, β-amyloid (Aβ) deposition, and abnormally phosphorylated Tau (Burns and Iliffe, 2009). However, its exact pathogenesis is yet to be elucidated.

There are many hypotheses on its pathogenesis, with the Aβ toxicity theory being widely recognized. However, it has met a lot of challenges, especially with several drug clinical trial failures (Joachim et al., 1989; Gotz and Ittner, 2008; Radde et al., 2008; Kumar et al., 2016). More recently, there has been an emphasis on the roles played by pathogenic microorganisms and the immune system in AD pathogenesis (Readhead et al., 2018; Dominy et al., 2019; Wu et al., 2019). Bacterial infections have shown a strong positive association with AD (Wallin et al., 2012; Bu et al., 2015; Maheshwari and Eslick, 2015; Dominy et al., 2019). At the initial stage, AD manifests as mild memory loss, which is directly associated with synapse loss and neuron dysfunction (Terry et al., 1991; Selkoe, 2002; Dubois et al., 2007). Moreover, it has been reported that the classical complement cascade drives the early synapse loss in AD (Hong et al., 2016).

Lipopolysaccharides (LPS) are the main cell wall constituents of Gram-negative bacteria, and are among the pathogen-associated molecular patterns recognized by the innate immune system to induce an *in vivo* immune response. LPS has been reported to induce memory impairment in rats (Zakaria et al., 2017). Besides, Hugh Perry and colleagues for example, have published extensively about the effects of LPS on learning and memory (Cunningham et al., 2005). Therefore, we hypothesized that *in vivo* injection of LPS could induce early memory impairment in mice similar to that in AD, with the immune system being involved. To examine this hypothesis, we established an AD mouse model, assessed the changes in learning and memory ability, and explored the pathological mechanisms involved.

## Materials and Methods

### Materials

The following are the details on the materials used for this study and where they were purchased from: equipment and software for the Morris water maze test (Shanghai Jiliang Technology Co., Ltd.) Reagents information is listed in Table 1.

**TABLE 1:** List of laboratory reagents.

**Reagents**	**Information**
Lipopolysaccharide	L2880, Sigma-Aldrich
TRIzol^TM^ reagent	15596018, Invitrogen^TM^
PrimeScript^TM^ RT reagent kit with gDNA eraser (perfect real time)	RR047A, Takara
TB green^®^ premix Ex Taq^TM^ II (Tli RNaseH Plus)	RR820A, Takara
BCA PROTEIN ASSAY KIT	23227, Thermo scientific^TM^
LDS sample buffer	84788, Thermo scientific^TM^
Gel handcast bundle A	HC1000R, Thermo scientific^TM^
TNF alpha mouse ELISA Kit	88-7324-22, Invitrogen^TM^
IL-1 beta mouse elisa Kit	88-7013-22, Invitrogen^TM^
IL-6 mouse elisa kit	88-7064-22, Invitrogen^TM^
Anti-Iba1, Rabbit	019-19741, Wako
Anti-PSD-95, mouse	MA1-046, Thermo scientific^TM^
Anti-synaptophysin, rabbit	MA5-14532, Thermo scientific^TM^
Anti-cleaved caspase-3, rabbit	9664S, CST
Anti-C1q, mouse	ab71940, Abcam
Anti-C3, mouse	ab11871, Abcam

### Animals

Male and female Kunming mice, 7–8 weeks old, were obtained from Guangdong Medical Laboratory Animal Center (Guangzhou, China). The animals were housed in a room with good ventilation, controlled temperature and humidity (20∼26°C, 60 ± 10%), and a 12 h/12 h circadian rhythm. The animals had free access to water and standard growing feed. The experimental procedures were performed in accordance with the Laboratory animals -General requirements for animal experiment and were approved by the Institutional Animal Care and Use Committee of Institute of Laboratory Animal Science, Jinan University.

The animals were randomly divided into two groups: control and LPS. The LPS group was intraperitoneally administrated with LPS (10 mg/kg), dissolved in normal saline, for 7 days. The control group was given the same volume of normal saline. Animals were kept warm after each administration.

In addition to 10 mice in each group for behavior test, 12 mice were sacrificed at d1 and 12 mice were sacrificed at d7 following LPS injection for histology and biochemical testing. After anesthesia with 1.2% Avertin, mice were perfused with pre-cooled normal saline. The tissue used for Western blot and qPCR was obtained directly. While tissue used for histology was obtained after continuous perfusion with 4% PFA, and then it was soaked with 4% PFA overnight and dehydrated with 30% sucrose solution.

### Morris Water Maze

After the mice were injected with LPS for 7 days, they were subjected to the Morris water maze test to evaluate whether LPS had damaged the learning and memory ability of the mice. This test involves a behavioral task and is most commonly used to determine the hippocampus-dependent spatial learning and memory ability of mice. We conducted the test as previously described (Morris, 1984; Vorhees and Williams, 2006) with appropriate adjustments. Briefly, a pool (120 cm in diameter) was evenly divided into four quadrants and filled with water to a 30 cm depth. We placed a platform (10 cm in diameter) in one of the four quadrants at about 1 cm below the water surface to hide it. We maintained the water temperature (22°C), room temperature and humidity (25°C, 60–80%), and the location of all objects in the room. Mice were forced to swim to find the hidden platform, starting from all four different quadrants, each day for 5 days. The time was recorded if they could find the hidden platform in 60 s, if not, there were guided toward the platform and allowed to stand on it for 10 s. After the 5 days, the platform was removed to test the mice.

### qPCR Assay

Total RNA of whole brain tissue was extracted using TRIzol^TM^ reagent, and the concentration and purity were assessed using a microvolume spectrophotometer. Next, the RNA was reverse transcribed (RT-PCR) to obtain cDNA for real-time fluorescent quantitative PCR (qPCR). RT-PCR and qPCR were conducted using RT reagent Kit (Takara) and TB Green (Takara) as per manufacturer instructions. The relative gene expressions of TNF-α, IL-1β, IL-6, C1qa, C3, and NPTX1 were computed from the qPCR data using the 2^–Δ^ ^Δ^ ^Cq^ method and normalized to β-actin. The primers used are listed in Table 2.

**TABLE 2:** Primers used in qPCR.

**Primers**		**Sequence (5′→ 3′)**
*Actb*	Forward	GGCTGTATTCCCCTCCATCG
	Reverse	CCAGTTGGTAACAATGCCATGT
*Tnf*	Forward	CCCTCACACTCAGATCATCTTCT
	Reverse	GCTACGACGTGGGCTACAG
*Il1b*	Forward	GCAACTGTTCCTGAACTCAACT
	Reverse	ATCTTTTGGGGTCCGTCAACT
*Il6*	Forward	TAGTCCTTCCTACCCCAATTTCC
	Reverse	TTGGTCCTTAGCCACTCCTTC
*NPTX1*	Forward	CCCGCTTCATCTGCACTTC
	Reverse	TCAGCTCCCTGATGGTCTCC

### Enzyme-Linked Immunosorbent Assay

The whole brain tissue was homogenized in phosphate-buffered solution (PBS) added with protease inhibitors, centrifuged, and the supernatant collected. Next, we added the supernatant to the enzyme-linked immunosorbent assay (ELISA) plates to assay the protein expression levels of TNF-α, IL-1β, and IL-6 in the brain according to the manufacturer’s instruction.

### Western Blot

The whole brain tissue was homogenized in RIPA lysis buffer. Next, the tissue homogenate was centrifuged and the concentration of total protein determined by the BCA method. The supernatant was added to loading buffer (4×) and boiled to obtain western blot (WB) samples. WB Samples were loaded on the prepared SDS-PAGE gel. After electrophoresis, the separated proteins in the gel were transferred to a PVDF membrane. The membrane was blocked with 5% skim milk at room temperature (RT) for 1 h, and subsequently incubated overnight with antibodies of C1q (1:500, Abcam) at −4°C overnight. The membrane was then washed in *Tris*-buffered solution with 0.1% Tween-20 (TBST) and incubated with HRP-conjugated goat IgG against mouse (1:2000, Earthox) at RT for 2 h. Finally, the membrane was washed, immersed in chemiluminescent HRP substrate, and the signals detected and photographed using an imaging system. Subsequently, the membrane was incubated again with β-actin (1:1000, Earthox) at −4°C overnight. The measures above were then repeated but the secondary antibody was replaced with HRP-conjugated goat IgG against rabbit (1:2000, Earthox).

### Immunofluorescence Assay

Brain tissues were fixed in 4% PFA, embedded with OCT, frozen on a flat surface of dry ice, and stored at −80°C. Next, the tissues were cryosectioned into 15 μm sections and mounted on glass slides. The sections were then blocked and permeated with 0.5% BSA with 0.05% Triton X-100 at RT for 2 h and were later incubated 16–24 h with antibodies of the proteins of interest at −4°C (synaptophysin 1:250, ThermoFisher; PSD-95 1:200, ThermoFisher; cleaved caspase 3 1:800, CST; C1q 1:50, Abcam; C3 1:100, Abcam; MAP2 1:200, Abcam; IBA1 1:1000, Wako). The next day the sections were incubated for 2 h with fluorescent dye-labeled antibody (Cy3 1:400, Earthox; 488 1:400, Earthox) at RT in darkness. The signals from the immunostained sections were then detected and photographed using a laser scanning confocal microscope. The synapses were quantified using a previously described method (Ippolito and Eroglu, 2010). The analysis process used a plug-in developed by Bary Wark from Duke University based on Image J software used for colocalization analysis.

### Statistical Analysis

Statistical analysis of data from the Morris water maze test was performed by two-way ANOVA for repeated measurements. We used *t*-test analysis to compare two independent sample groups and one-way ANOVA to compare three or more sample groups. Data were presented as mean ± S.E.M. *P* < 0.05 was considered as statistically significant.

## Results

### Peripheral Injection of LPS Induces Learning and Memory Impairment in Mice

**FIGURE 1:**
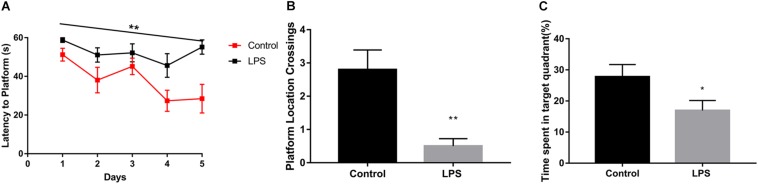
Effects of LPS on memory acquisition and retention performance in the Morris water maze behavioral task. After 7 days of LPS administration, the control and LPS-injected mice were subjected to the Morris water maze spatial learning task for 5 days. **(A)** Latency before finding the target platform was recorded daily (mean of 4 trials per day, ^∗∗^*P* < 0.01 by repeated measures two-way ANOVA, *n* = 10). **(B,C)** After 5 trial days, the control and LPS-injected mice were tested and assessed based on the number of times they crossed the platform location **(B)** and the time spent in the target quadrant(C). ^∗^*P* < 0.05, ^∗∗^*P* < 0.01 vs. control by unpaired two-tailed *t*-test, *n* = 10.

We found that LPS-injected mice required more time to locate the hidden platform (Figure 1A). Moreover, after the 5-day training period and removal of the platform, LPS-injected mice crossed the area where the platform had been located significantly fewer times than the controls (Figure 1B). In addition, they spent less time in the quadrant where the platform had been located (Figure 1C).

### Peripheral Injection of LPS Causes Hippocampal Neuronal Impairment

**FIGURE 2:**
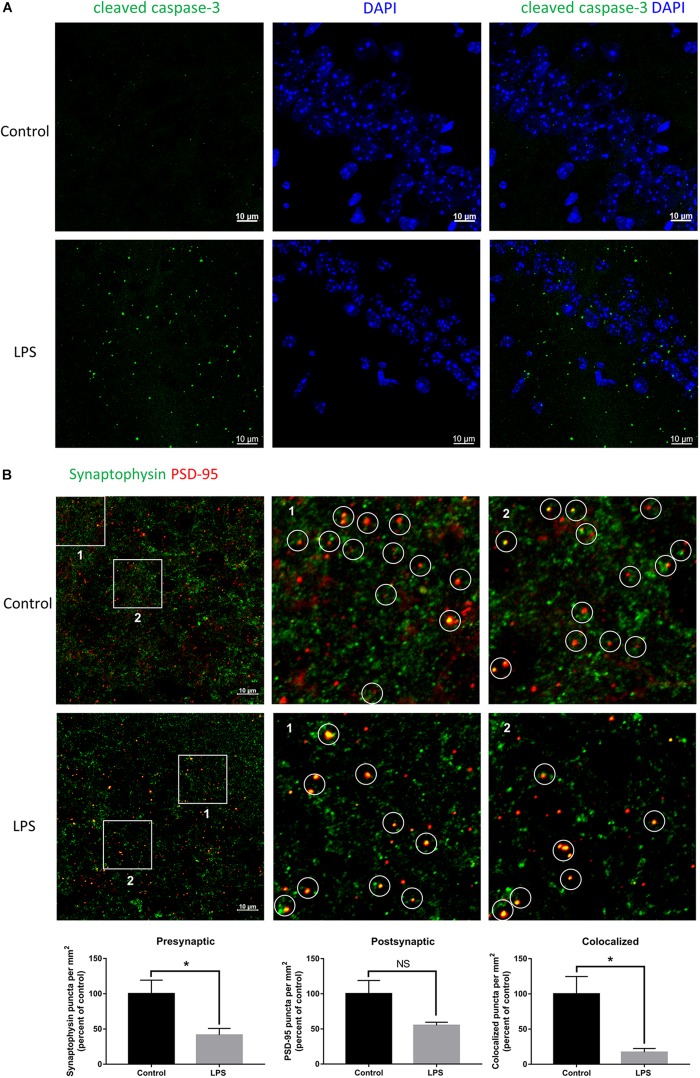
Effects of LPS on neurons and synapses in the hippocampus. **(A)** Immunostaining of neurons with cleaved caspase-3 at the hippocampal CA1 region of the control and LPS-injected mice. DAPI was used to stain the nucleus. **(B)** Immunostaining and quantification of colocalized presynaptic and postsynaptic puncta using synaptophysin and PSD-95 as the markers, respectively, at the hippocampal CA3 region of the control and LPS-injected mice. ^∗^*P* < 0.05, NS, no significant, by unpaired two-tailed *t*-test, *n* = 5.

Given the crucial role of the hippocampus in the spatial memory in mice, we performed immunofluorescence staining of this brain region to verify whether biochemical changes occurred from its impairment. LPS-injected mice showed significant positive staining for cleaved caspase-3 within the hippocampal CA1 region (Figure 2A), which indicated that neurons had been damaged and were undergoing apoptosis. Moreover, we quantified the number of synaptic terminals of hippocampal neurons (Figure 2B). Both the number of presynaptic puncta and colocalized presynaptic and postsynaptic puncta were decreased in LPS-injected mice compared with the controls, indicating loss of synapses within the hippocampal CA3 region. These results implied that peripheral injection of LPS induced damage of hippocampal neurons and their synapses.

### Peripheral Injection of LPS Causes Inflammation in the Brain

**FIGURE 3:**
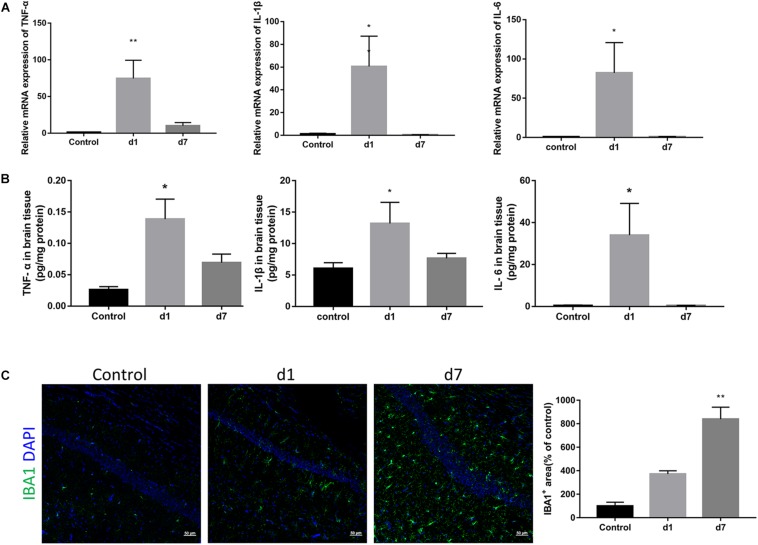
Effects of LPS on the expression of pro-inflammatory cytokines and microglia in the brain. **(A)** The relative mRNA expression of proinflammatory cytokines TNF-α, IL-1β, and IL-6 in the brains of the control and LPS-injected mice were determined by qPCR assay (*n* = 6). **(B)** Protein levels of TNF-α, IL-1β, and IL-6 in brain homogenate from the two groups were determined by ELISA (*n* = 4). **(C)** Immunostaining and relative quantification of IBA1 specifically expressed in macrophages/microglia. Brain tissues were obtained from the control mice and mice administered with LPS on the first day and after 7 days (*n* = 3). ^∗^*P* < 0.05, ^∗∗^*P* < 0.01 vs. control, by one-way ANOVA.

To elucidate the underlying mechanism of synaptic loss, we first determined whether LPS could activate an immune response in the brain. As shown in Figure 3, the mRNA expression of proinflammatory cytokines in the brain, TNF-α, IL-1β, and IL-6, increased rapidly after a single injection of LPS, and then gradually decreased with subsequent injections (Figure 3A). Protein expressions of the three cytokines were consistent with their corresponding mRNA expressions (Figure 3B). IBA1 immunostaining indicated a significant increase and activation of microglia within the hippocampi of LPS-injected mice (Figure 3C).

### Peripheral Injection of LPS Induces Activation of the Classical Complement Pathway in the Brain

**FIGURE 4:**
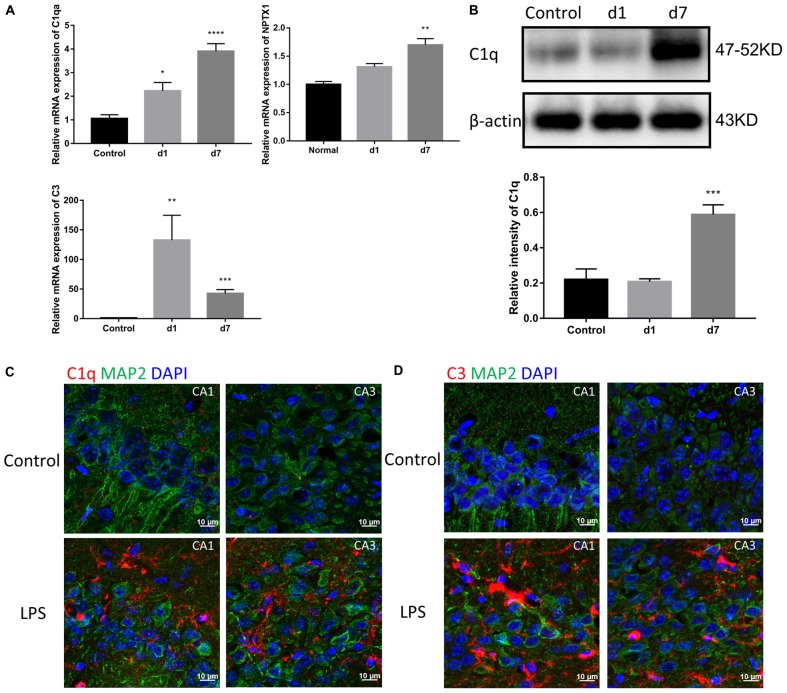
Effects of LPS on the expression of complements in the brain. **(A)** The relative mRNA expression of C1qa, C3, and NPTX1 in the brains of the control mice and mice treated with LPS for 1 or 7 days were determined by qPCR assay. *NPTX1* encodes neuronal pentraxin 1, which is a homogenous acute-phase protein synthesized in the brain. Data were presented as means ± SEM (^∗^*P* < 0.05, ^∗∗^*P* < 0.01, ^∗∗∗^*P* < 0.001, ^∗∗∗∗^*P* < 0.0001 vs. control, by one-way ANOVA, *n* = 6). **(B)** Relative protein levels of C1q were determined by Western blot. The intensity of the blots was calculated using *Image J* and data were presented as mean ± S.E.M. (^∗∗∗^*P* < 0.001 vs. control, by one-way ANOVA, *n* = 4). **(C,D)** Immunostaining of C1q and C3, respectively, in both the hippocampal CA1 and CA3 regions. MAP2 was used as the neuron marker.

Considering the detected increase in the expression of TNF-α, IL-1β, and IL-6, we assessed the levels of the first two classical complement pathway components. Both mRNA and protein expression of C1 and C3 in the brain were increased in the mice injected with LPS (Figures 4A,B). Immunostaining of the hippocampal CA1 and CA3 regions showed a significant increase in C1q and C3 around neurons in LPS-injected mice (Figures 4C,D).

### A Complements-Microglia Axis Mediates Synapse Impairment

**FIGURE 5:**
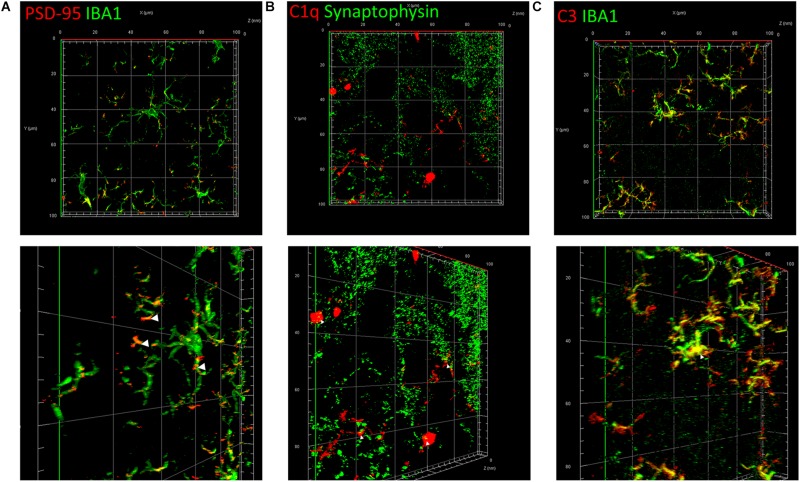
Classical complements and microglia mediate the impairment and loss of synapses. **(A)** Immunostaining of IBA1, a specific microglia marker, and PSD-95, a postsynaptic marker. **(B)** Immunostaining of C1q and the presynaptic marker synaptophysin. **(C)** Immunostaining of IBA1 and C3. The picture in the lower row of each group is the shot of the corresponding three-dimension view after appropriate rotation.

To elucidate the common mechanism behind the synapse loss after LPS injection, we analyzed the interrelationship between synapse loss, complements, and microglia. Fluorescent double staining displayed a significantly higher volume of internalized PSD-95 in microglia in the hippocampus of LPS-injected mice compared with that in the controls, indicating that LPS induced microglial engulfment of synapse elements (Figure 5A and Supplementary Video S1). Colocalization of C1q and presynaptic puncta revealed a connection between synapse loss and complements (Figure 5B). Moreover, colocalization of C3 and microglia indicated a connection between complements and microglia (Figure 5C). These results suggest that complements and microglia formed an axis to attract the phagocytic microglia to engulf the “marked” synapses.

## Discussion

In this study, we found that peripheral injection of LPS induced learning and memory impairment in mice. LPS used to be thought to induce AD via Aβ deposition (Lee et al., 2008), but recently some controversies were surrounded with it – Aβ may act as an antimicrobial peptide in bacterial infection (Kumar et al., 2016). In this study, we aimed to ascertain a model of memory loss in WT mice through repeated dosing with LPS, and preliminarily explore whether the immune system plays a role in this process. Our further examination revealed that this impairment was attributed to the microglia-induced synapse damage. Many studies have reported that complements and microglia are involved in synaptic pruning during brain development (Stevens et al., 2007; Schafer et al., 2012; Bialas and Stevens, 2013). C1q, the initiating protein in the classical complement pathway, is reported to be localized to the selected synapses to mediate their specific pruning. We showed that immune activity involving activation of the classical complement cascade in the brain mediated the microglial phagocytosis of synapses. Complements, which form part of the innate immune system, can be activated by immune complexes, apoptotic cells, and some antigens. They are upregulated by some cytokines, including interferons, interleukins, and tumor necrosis factors during the acute phase reaction. Some of the cytokines are suppressed after activating the complement cascade. Microglia are the major resident immune cells of the central nervous system (CNS) and continuously detect, survey, transmit, and respond to extracellular signals to maintain CNS homeostasis (Salter and Stevens, 2017), and can be activated when CNS inflammation emerges. In our study, we found that during memory impairment in mice, inflammatory factors first increased significantly, and then decreased gradually with the activation of complement system. Meanwhile, microglia aggregated. Our findings are consistent with those of recent studies on the immune system and AD. The immune system plays an important role in other acute and chronic brain disorders, such as Huntington’s disease, amyotrophic lateral sclerosis, multiple sclerosis, Parkinson’s disease, and AD. Complements are downregulated in the CNS of normal adults, but have been reported to be aberrantly activated in neurodegenerative diseases, such as glaucoma and AD (Stevens et al., 2007; Veerhuis et al., 2011). Similar to during postnatal development, C1q has been reported to be elevated in specific brain regions in early stage AD, prior to the formation of overt plaques, which promotes a CR3-dependent microglial engulfment of synapses (Hong et al., 2016). During early apoptosis, C1q, together with pentraxins, opsonize the apoptotic cells to enhance their clearance by microglia (Nauta et al., 2003; Fraser et al., 2010). In this study, we detect an activation of immune system in the brain after peripheral administration of LPS. During the process, complements- C1q and C3, as well as microglia have significantly increased in hippocampus and tightly connected to synapses.

However, since LPS did not enter the brain, it is still unclear how the molecules stimulated the CNS immune system (Banks and Robinson, 2010; Abbott, 2018). Some studies have offered hypothesis for the mechanisms involved, with many of them suggesting that LPS can stimulate the peripheral immune system to produce large amounts of pro-inflammatory cytokines and amyloid-beta protein (Aβ), which can both pass through the blood-brain barrier and enter the brain (Pan and Kastin, 2002; Evin et al., 2003; Bu et al., 2018). Moreover, it is clear that these two molecules stimulate the CNS immune system. Bu et al. (2018) suggested that AD is induced by blood-derived-Aβ. In future studies, we can give the mouse an exogenous injection of labeled cytokines or Aβ after LPS to directly detect whether they do enter the brain in the process. Whether synaptic debris exists in the lysosome of microglia and the exact mechanism how C3 urge microglia to close to C1q connected synapses is also an interesting idea to conduct follow-up research. In addition, considering the sensitivity to LPS and the consequent production of inflammatory cytokines differs hugely between human and rodent animals (Zakaria et al., 2017), the model needs to be further improved in terms of the administration mode and dose, animal species, and sex etc.

## Conclusion

In conclusion, our findings demonstrate that mice with LPS-induced learning and memory impairment could be an appropriate animal model for early memory impairment in AD. The immune system in CNS drives the synapse-loss caused impairment. Currently, we are screening AD early intervention drugs using this model. It helps us to focus on an isolated chemical monomer from medicinal plants, and prompts us to develop it as a drug or health supplement for early intervention in AD patients.

## Data Availability Statement

All datasets generated for this study are included in the manuscript/Supplementary Files.

## Ethics Statement

This study was carried out in accordance with the recommendations of Laboratory animals-General requirements for animal experiment, Institutional Animal Care and Use Committee. The protocol was approved by the Institutional Animal Care and Use Committee.

## Author Contributions

H-ML proposed the study concept and approved the final version of the manuscript. FX and WZ performed the pre-study of the topic. Y-RX participated in the whole work, including operating experiments, data analysis, and drafting the manuscript. J-XJ participated in the animal experiments. YH and J-PP assisted in the biochemical experiments. X-NM and QG prepared the necessary reagents and equipments.

## Conflict of Interest

The authors declare that the research was conducted in the absence of any commercial or financial relationships that could be construed as a potential conflict of interest.
