# Supplementary material for: The Immune System Drives Synapse Loss During Lipopolysaccharide-Induced Learning and Memory Impairment in Mice
Source: Front Aging Neurosci. 2019 Nov 15;11:279. doi: 10.3389/fnagi.2019.00279 (PMC6873885; doi:10.3389/fnagi.2019.00279)
Supplement: Supplementary file 2 [file Data_Sheet_1.docx]

Supplementary Material

## Supplementary Figures

**
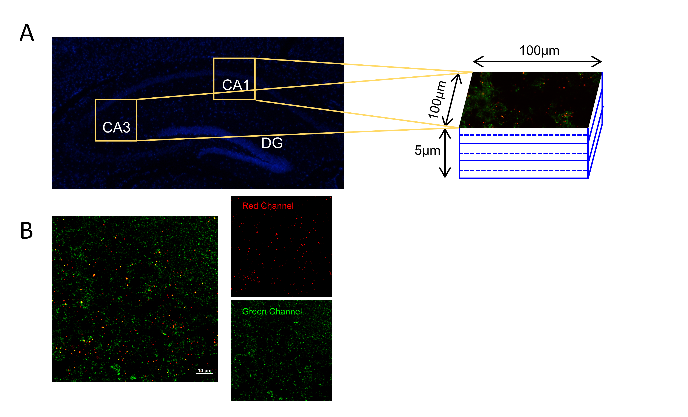
**

**Supplementary Figure 1.** **Schematic diagram of histology.** (A) In figure 1and 3, to standardize the quantification, we fixed the depth to 5 μm and the interval to 0.33 μm. In figure 4, we selected all C1q or C3 staining ranges from top to bottom in a 15-micron-thick brain tissue section, took a picture every 1 μm, and overlapped all the photos to get the final Z-stack image. In figure 5, to better analyze the interrelationship between microglia, we choose a complete view of microglia in depth, take a picture every 0.33 μm, and then build a 3D image. (B) To quantify different staining, we split the pictures to two staining channels. Analyze different channels separately and find the colocalization puncta using *Image J* plugin.

**
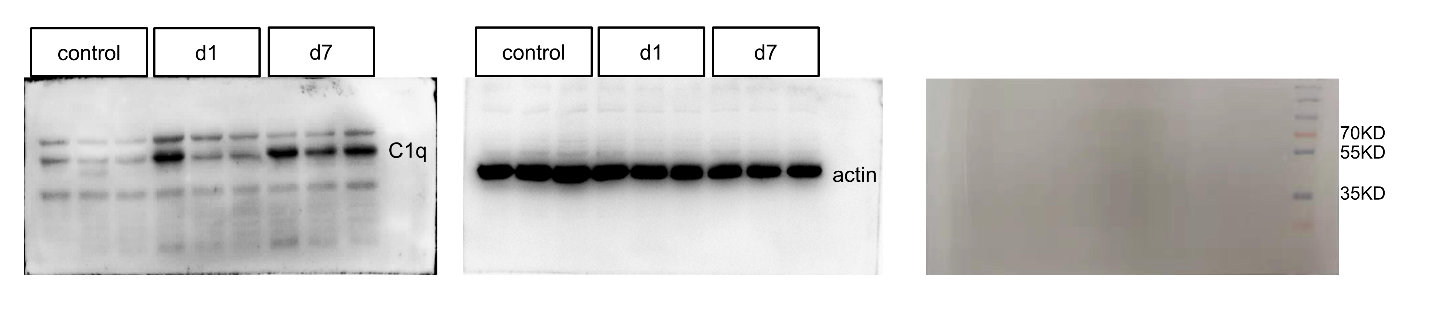
**

**Supplementary Figure 2.** **Whole gel figure of Western blot.** The three picture was obtained form the same PDVF membrane, and was incubated with anti-C1q antibody and anti-β-actin antibody separately.

## Supplementary Video

**Supplementary Video 1.** **Three-dimensional maps of complement, microglia and synapse staining.**
